# When the Clock Is Ticking: The Role of Mitotic Duration in Cell Fate Determination

**DOI:** 10.1002/bies.70061

**Published:** 2025-08-30

**Authors:** Cornelia Sala, Elmar Schiebel

**Affiliations:** ^1^ Zentrum Für Molekulare Biologie Der Universität Heidelberg (ZMBH), Deutsches Krebsforschungszentrum (DKFZ)‐ZMBH Allianz Universität Heidelberg Heidelberg Germany

## Abstract

Mitosis is a crucial phase of the cell cycle, during which several mechanisms work together to ensure accurate chromosome segregation and to eliminate defective cells if errors occur. One key mechanism is the spindle assembly checkpoint (SAC), which upon mitotic errors—such as those induced by genetic mutations, drug treatments, or environmental stresses—arrest cells in mitosis. Arrested cells may undergo apoptosis during mitosis or eventually exit mitosis even if the damage remains unrepaired. Mitotic exit is driven by a reduction in cyclin B1 levels, regulated during mitosis by multiple mechanisms affecting both its synthesis and degradation. Strikingly, cells harboring the tumor suppressor p53 can monitor the duration of mitosis and encode this information as a form of “mitotic memory”. This memory influences the fate of daughter cells after mitotic exit by inducing G1 arrest through p53‐dependent expression of the cyclin‐dependent kinase (CDK) inhibitor p21. Recent studies have proposed mechanisms by which cyclin B1 levels are regulated during mitotic arrest and how p53 promotes mitotic‐arrest‐dependent transcription of p21 in G1. These findings indicate that both the expression of regulators that control mitotic duration and the activity of proteins that monitor the duration of mitosis and halt proliferation work together to determine cell fate following mitotic errors. Understanding these mechanisms offers valuable insights for cancer therapy, particularly regarding the strategic application of antimitotic agents.

## Graphical abstract

Recently, several studies identified mechanisms by which cells measure mitotic duration and how this influences cell fate, leading to normal cell cycle progression or G1 arrest. In light of drug‐resistant cancer cells that continue proliferating even after mitotic errors, understanding these mechanisms opens the door for new potential therapeutic approaches.

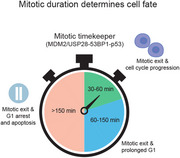

## 1 Introduction

At the onset of mitosis, spindle microtubules attach to the kinetochores of sister chromatids, which remain connected by the ring‐like cohesin complex. These kinetochore–microtubule interactions are dynamically regulated until each sister kinetochore is stably linked to microtubules from opposite spindle poles—a configuration known as biorientation [1].

The spindle assembly checkpoint (SAC) is a kinetochore‐based surveillance mechanism that monitors kinetochore–microtubule attachments [2]. During spindle assembly in prometaphase, not all kinetochores are attached to microtubules, and therefore the SAC is active and promotes the assembly of the mitotic checkpoint complex (MCC) consisting of the proteins MAD2, BUB3, BUBR1, and CDC20. The MCC then inhibits the anaphase‐promoting complex/cyclosome (APC/C) [3], which is an E3 ubiquitin ligase that normally targets key regulatory proteins for degradation by the proteasome, including cyclin B1 and securin. Cyclin B1 and securin act as inhibitors of the protease separase, which cleaves the cohesin complex. By inhibiting separase, these proteins help maintain the cohesion between sister chromatids. However, as soon as biorientation is achieved, the SAC is silenced, promoting MCC disassembly and activation of the APC/C [4]. This triggers the degradation of cyclin B1 and securin, activating separase to cleave the cohesin complex, while the decline of cyclin B1 drives mitotic exit. As a result, the mitotic spindle separates the sister chromatids during anaphase, moving them toward opposite spindle poles [5].

**FIGURE 1:**
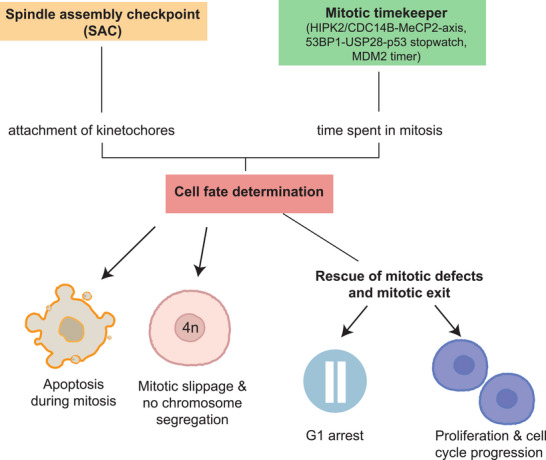
Cell fate determined by the spindle assembly checkpoint and the mitotic timekeeper mechanisms. The spindle assembly checkpoint (SAC) is a surveillance system, ensuring proper mitotic progression. Prolonged SAC activity, due to unattached kinetochores, arrests cells in mitosis. Mitotically arrested cells can undergo several distinct fates: (1) apoptosis during mitosis, resulting in cell death; (2) mitotic slippage, in which cells exit mitosis without proper chromosome segregation or cytokinesis, leading to polyploidy; or (3) successful chromosome segregation, cytokinesis, and exit into the G1 phase. G1‐phase cells can either halt proliferation or continue progressing through the cell cycle.

Mitotic errors, such as those caused by anti‐cancer drugs or cellular stress, can lead to prolonged mitotic arrest due to sustained SAC activation, resulting from defective kinetochore–microtubule attachments. Cells arrested in mitosis face several possible fates (Figure 1), depending on the duration of the arrest and their p53 status. Recent studies have begun to uncover the molecular mechanisms by which cells are maintained in mitosis in response to persistent SAC activation, how cells measure mitotic duration, and how these processes influence subsequent cell fate decisions.

## 2 Keeping Cells in Mitosis

In normally cycling cells, the SAC is active from prophase until metaphase, when all kinetochores are correctly attached to the spindle microtubules—a process that in most cultured cells typically lasts 30–60 min [6]. When spindle defects occur, the SAC remains active, with the MCC inhibiting the APC/C [7]. This results in persistent high cyclin B1 levels and cell cycle arrest in prometaphase. Cells that remain arrested in mitosis for an extended period may undergo cell death through p53‐independent mechanisms (Figure 1). Such mechanisms involve either the survival‐promoting protein MCL1 [8] or the cGAS‐STING pathway [9], and both induce apoptosis in a mitosis‐duration‐dependent manner. Alternatively, cells can exit mitosis and enter the G1 phase through APC/C‐mediated degradation of cyclin B1, which occurs once the SAC is silenced. Another possibility is that, despite an active SAC, cyclin B1 levels fall below a critical threshold, allowing cells to progress into G1 [7] (Figure 1). In the most extreme case, known as mitotic slippage, cells fail to properly segregate their chromosomes and bypass cytokinesis, resulting in the formation of polyploid cells. Therefore, maintaining high cyclin B1 levels in cells with mitotic defects is crucial to ensure sustained prometaphase arrest and the subsequent elimination of defective cells.

**FIGURE 2:**
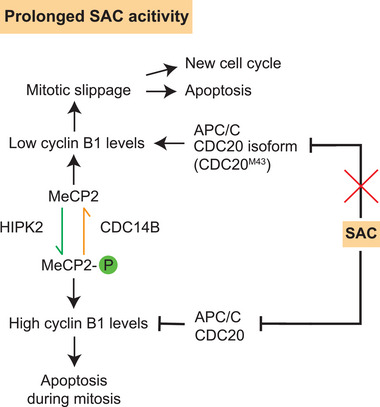
Pathways modulating cyclin B1 levels during prolonged spindle assembly checkpoint (SAC) activation. Phosphorylated methyl‐CpG‐binding protein 2 (MeCP2) promotes the translation of cyclin B1, thereby sustaining high cyclin‐dependent kinase (CDK)1–cyclin B1 activity during mitotic arrest. The kinase homeodomain‐interacting protein kinase 2 (HIPK2) phosphorylates MeCP2 at Ser92, while the phosphatase CDC14B counteracts this modification. Additionally, during mitotic arrest, translation of the anaphase‐promoting complex/cyclosome (APC/C) substrate adaptor CDC20, responsible for targeting cyclin B1 for degradation, is initiated from the M43 start codon. The CDC20^M43^ isoform supports cell proliferation but cannot sustain SAC‐induced mitotic arrest, meaning that even under persistent SAC activation, APC/C–CDC20^M43^ degrades cyclin B1. Decreasing cyclin B1 levels by an increase in CDC14B or accumulation of CDC20^M43^ promotes mitotic slippage into G1.

How do cells arrested in mitosis regulate cyclin B1 levels? Contrary to earlier assumptions that protein translation is largely suppressed during mitosis, recent evidence indicates that translation of some mRNAs does occur during this phase [10], including for cyclin B1 [11]. A pathway involving homeodomain‐interacting protein kinase 2 (HIPK2), CDC14B phosphatase, and methyl‐CpG‐binding protein 2 (MeCP2) has been shown to enhance the SAC‐induced mitotic arrest by promoting cyclin B1 translation (Figure 2) [12]. HIPK2, a stress‐responsive kinase [13], phosphorylates MeCP2, a chromatin‐associated factor known for its role in neural development and Rett syndrome [14]. Phosphorylation of MeCP2 at serine 92 promotes the translation of cyclin B1, potentially by interacting with the 5′ untranslated region (UTR) of its mRNA. CDC14B counteracts this by dephosphorylating MeCP2 [12]. In line with these findings, deletion of *CDC14B* enhances mitotic arrest by permitting cyclin B1 translation, while its overexpression facilitates mitotic exit in cells experiencing prolonged SAC activation. The interplay between the key regulators HIPK2 and CDC14B governs cyclin B1 translation in mitotically arrested cells, thereby influencing cell fate decisions.

In addition, alternative translation start sites of CDC20 were found to modulate the duration of mitotic arrest [15]. Leaky ribosome scanning produces a shorter CDC20 isoform (CDC20^M43^) that supports cell proliferation but cannot sustain SAC‐mediated mitotic arrest in response to the kinesin motor protein Eg5/KIF11 inhibitor S‐trityl‐l‐cysteine (STLC), which prevents the formation of a bipolar mitotic spindle. The shorter CDC20^M43^ proteoform accumulates during mitotic arrest and promotes mitotic slippage by inducing a decline in cyclin B1 levels.

Taken together, these findings indicate that mitotic duration is determined by multiple factors, including SAC robustness, the interplay between CDC14B and HIPK2, and CDC20^M43^ (Figure 2). CDC14B and HIPK2 regulate via MeCP2 phosphorylation translation of cyclin B1 while CDC20^M43^ promotes cyclin B1 degradation by the APC/C even when the SAC is active. This leads to mitotic slippage of cells with an active SAC and thus promotes the escape from mitosis‐dependent cell death pathways.

## 3 The Mitotic Timer Regulates p53

Doxsey and coworkers were among the first to demonstrate that loss of centrosome integrity resulting in mitotic defects triggers a p53–p21‐dependent arrest at the G1–S transition [16]. They proposed a novel checkpoint linking centrosome function to cell cycle progression. Subsequent studies showed that prolonged prometaphase induced by treatment with microtubule poisons followed by washout can arrest daughter cells in G1 via a p53‐dependent mechanism, despite successful completion of the previous mitosis [17]. Finally, long‐term measurements indicated that cells arrested in G1 ultimately underwent apoptosis, leading to their elimination [18]. Together, these striking findings suggest that, even after satisfaction of the SAC, mechanisms exist that monitor the duration of mitosis and can halt cell‐cycle progression in G1 if mitosis is prolonged. Notably, it appears that the length of mitosis—rather than the underlying cause of the delay—triggers the p53 response.

The discovery of a mitotic‐duration–dependent G1 arrest raises the question of how cells measure mitotic duration and transmit this information to the cell cycle machinery. A first clue toward answering this question emerged from genome‐wide CRISPR/Cas9 screens for genes conferring resistance to centrinone, a drug that inhibits Polo‐like kinase 4 (PLK4), the master regulator of centriole duplication. Centrinone treatment leads to reversible centrosome loss over multiple cell divisions, resulting in mitotic delay due to defective spindle formation and eventually p53‐dependent G1 arrest [19, 20, 21]. These screens not only identified *TP53* (coding for p53) but also genes encoding the p53‐binding protein 53BP1 and the deubiquitinase USP28 [19, 22, 23]. In these knockout cell lines, prolonged mitosis by centrinone was tolerated, as the mitotic timing mechanism was disrupted and an acentrosomal pathway took over to enable bipolar spindle assembly.

Two recent publications have now significantly advanced our understanding of how cells measure mitotic duration and convey this information to cell cycle regulators. Meitinger et al. [6] provided evidence for a staged cellular response to the length of mitosis. In nontransformed, telomerase‐immortalized retinal pigment epithelial‐1 (RPE1) cells, a normal mitotic duration of 30–40 min elicited only low p21 expression in G1. Extension of mitosis to 60–150 min resulted in a longer G1 phase due to increased—but not sustained—p21 levels. However, when mitotic duration exceeded a threshold of 150 min, p21 expression in G1 persisted, and cells failed to re‐enter the cell cycle.

**FIGURE 3:**
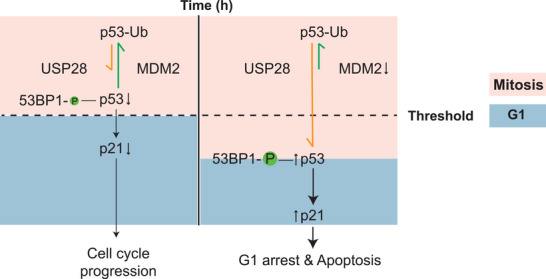
p53 levels modulated by the MDM2 mitotic timer and the 53BP1–USP28–p53 mitotic stopwatch. The interplay between the ubiquitin ligase MDM2 and the deubiquitinase USP28 modulates p53 levels. Cells with normal mitotic duration show low levels of phosphorylated 53BP1 and p53, thereby preventing p21 expression and promoting proliferation and cell cycle progression. However, if the time spent in mitosis exceeds a certain threshold, the amount of phosphorylated 53BP1 increases, which can then bind to p53 and USP28. p53, in turn, accumulates through USP28‐mediated deubiquitination, while MDM2 levels decrease due to its short half‐life and lack of expression in mitosis. The increase in p53 levels promotes p21 expression in G1 and leads to a G1 arrest and eventually apoptosis.

How does this mechanism operate at the molecular level? Meitinger et al. [6] identified a “stopwatch complex”—comprising 53BP1, USP28, and p53—encoded by genes previously detected in their centrinone‐resistant screen [21], as essential for this timing function (Figure 3). The formation of the complex is restricted to mitosis and correlated with the time cells spend in this phase. The authors found that Polo‐like kinase 1 (PLK1) promotes stopwatch complex formation by phosphorylating 53BP1, which then can bind to p53 and USP28. USP28 in the stopwatch complex likely deubiquitinates and stabilizes p53, which in the subsequent G1 phase can promote p21 transcription. Based on these results, the authors propose that mitotic duration influences the assembly of the stopwatch complex, and that p21 levels in G1 reflect this duration through the abundance of the stopwatch complex.

An alternative mechanism how cells measure the mitotic duration was proposed by Fulcher et al. [24], involving the E3 ubiquitin ligase MDM2 [25] (Figure 3), which plays an important role as a negative regulator of p53, keeping it at low levels by targeting it for degradation. How does MDM2 measure the duration of mitosis? Their data suggest that MDM2 undergoes self‐ubiquitination during both interphase and mitosis, facilitated by the E2 ubiquitin‐conjugating enzyme UBE2D, which ultimately leads to its degradation [24]. Due to its self‐destruction mechanism, MDM2 has a short half‐life of approximately 24–29 min. Since MDM2 is not translated during mitosis, its short half‐life leads to a progressive decline in MDM2 protein levels as the duration of mitosis increases. This property is exploited to measure the duration of mitosis. Although MDM2 levels decline, p53 accumulates accordingly, promoting the transcription of p21 during the G1 phase in a manner dependent on the duration of mitosis.

Supporting this model, a 1h mitotic delay in the presence of MDM2 led to a G1 duration of about only **∼**9.8 h. However, upon targeted depletion of MDM2 using the PROTAC degron system, a 1‐h mitotic arrest resulted in elevated p21 levels during the subsequent G1 phase and a G1 arrest for 19.8 h. Finally, overexpression of *MDM2* after a 4‐h mitotic arrest enabled cells to resume proliferation rather than remain arrested in G1. These findings strongly indicate that the MDM2 level is crucial for reporting mitotic duration and that elevated levels reduce the mitotic timer response.

Both upregulation of the PLK1–53BP1–USP28 “mitotic stopwatch” and downregulation of the MDM2 “mitotic timer” trigger p53‐dependent p21 expression in G1, leading to cell‐cycle arrest. Because measuring mitotic duration involves a ubiquitin ligase (MDM2) and a deubiquitinating enzyme (USP28) that ultimately regulate p21 levels, these two models may represent components of a single pathway. Acting in concert, they could fine‐tune the G1 arrest after mitotic delay, serving as a safeguard against proliferation following prolonged mitosis. In this model, the short half‐life of MDM2 allows it to function as an early sensor of extended mitosis, whereas the PLK1–53BP1–USP28 stopwatch accumulates more gradually to maintain elevated p53 during sustained mitotic arrest. Given that mitotic errors can have severe consequences, it is plausible that mitotic timing is regulated by parallel or overlapping mechanisms that may partially compensate for one another. Further experiments are needed to measure the relative importance of both mechanisms across different cell types and in response to various mitotic disturbances.

The studies by Meitinger et al. [6] and Futcher et al. [24] are significant for three reasons: first, they highlight the critical role of the mitotic timekeeper pathways, the stop watch complex and the mitotic timer, in halting cell proliferation in response to mitotic delays; second, they offer insights into mechanisms of cancer resistance to specific cytostatic drugs (see below); and third, restoring the mitotic timekeeper function by modulating the different key players may serve as a strategy to sensitize cancer cells to drugs that disrupt spindle formation.

## 4 An Echo of Mitotic Duration is Transferred to the Next Cell Cycle

Meitinger et al. [6] observed that cells possess a form of mitotic memory, integrating prolonged mitotic durations over multiple cell cycles. When cumulative mitotic duration exceeds a critical threshold, cells arrest in G1 due to p53‐mediated activation of p21. However, because MDM2, p53, and p21 all have short half‐lives—on the order of minutes—they are unlikely to serve as memory components for a mitotic timing mechanism that operates across a full 24‐h cell cycle. Instead, it is more likely that the memory resides in intrinsic properties of the 53BP1–USP28–p53 stopwatch complex itself.

During an unperturbed cell cycle, 53BP1 binds to chromatin in interphase, resulting in low levels of the stopwatch complex in interphase cells. In contrast, during prolonged mitosis, 53BP1 is gradually phosphorylated by PLK1, promoting assembly of the stopwatch complex. Due to PLK1's function as a key mitotic regulator and its high activity, it appears intriguing how phosphorylated 53BP1 accumulates slowly over time during extended mitosis. A recent study found that 53BP1 is gradually released from kinetochores during mitosis, after which the soluble form of 53BP1 is phosphorylated by PLK1. This sequence of events defines a specific time window in which 53BP1 is accessible to PLK1, thereby explaining the slow accumulation of phosphorylated 53BP1 [26]. Additionally, 53BP1 may be a poor substrate for PLK1, or its phosphorylation may depend on other factors influencing the reaction kinetics.

Interestingly, in *CDKN1A* (coding for p21)‐deficient cells, which fail to arrest in G1 after extended mitosis, the stopwatch complex formed during prolonged mitosis remains soluble and persists throughout all phases of the subsequent interphase, even without PLK1 activity, raising the question of how this persistence is maintained [6]. These findings suggest that the stopwatch complex is both stable and inherited by daughter cells, allowing it to integrate mitotic duration across successive cell cycles.

## 5 The Impact of Mitotic Duration and the Mitotic Timer on Cancer

Stopwatch‐positive cells monitor the duration of mitosis in response to disturbances and halt their proliferation accordingly. This stopwatch mechanism acts as a surveillance system, eliminating potentially dangerous cells before they can undergo malignant transformation. Consistently, the components of this mitotic stopwatch are considered as tumor suppressor genes [27]. Among them, *USP28* is particularly interesting, as it is suggested to have no critical function in the DNA damage response, unlike *TP53* and *TP53BP1*. Nevertheless, USP28 recruitment to DNA damage sites and effects on its activity after genotoxic stress has been shown [28, 29], leaving it an open question if, or to what extent, USP28 contributes to the DNA damage response.

In an analysis of 15 cancer cell lines expressing p53 to assess stopwatch function, a significant portion were found to be partially or completely stopwatch‐deficient due to mutations in either *TP53BP1* or *USP28*. Other cell lines lacking the stopwatch function harbored mutations that hyperactivated the phosphatase WIP1. Since WIP1 antagonizes p53, treatment with a WIP1 inhibitor partially restored stopwatch function in these cell lines [6]. However, a recent study came to a different conclusion, showing that RPE1 cells expressing a truncated *WIP1* version that shows gain‐of‐function are still sensitive to mitotic delay and arrest in G1 [30]. Furthermore, cell lines harboring natural gain‐of‐function mutations in *WIP1* (such as U2OS and HCT116) showed no increase in G1 arrest after a mitotic delay when *WIP1* was knocked out [30]. The authors show that cancer‐associated activating mutations in WIP1 increase the threshold for DNA damage signaling. As a result, G2‐phase cells harboring DNA damage can progress into mitosis while maintaining elevated MDM2 levels, which subsequently inhibit the mitotic‐timer‐dependent G1 cell‐cycle arrest.

Meitinger et al. [6] also highlight that the functional status of the stopwatch in cancer cells influences their response to the PLK4 inhibitor centrinone. Stopwatch‐competent cancer cell lines halt proliferation in response to centrinone, whereas stopwatch‐deficient cell lines showed an improved proliferation rate. These findings were confirmed using Taxol, an anti‐cancer drug that disrupts microtubule dynamics, and GSK92329, a clinically approved inhibitor of the kinesin motor protein CENP‐E, which is essential for chromosome segregation during mitosis. These data indicate that loss of stopwatch function will lead to resistance against cancer drugs, while restoring this function may recover their effectiveness.

An additional therapeutic approach involves targeting MDM2. Amplification of *MDM2* is commonly observed in sarcomas, as well as in other cancer types such as breast and colorectal cancer [31, 32, 33]. In light of the recent findings suggesting a critical role of MDM2 in measuring the mitotic duration, this opens new insights of how the mechanics in *MDM2* overexpressing cancer cells lead to uncontrolled proliferation and anti‐mitotic drug resistance. Indeed, Fulcher et al. [24] showed that overexpression of *MDM2* overrides the effect of an extended mitosis, which otherwise results in a G1 arrest. Therefore, these *MDM2* overexpressing cells evade the G1 arrest in response to mitotic poisons and continue cell cycle progression after exiting mitosis.

Both mitotic timekeeper mechanisms are p53‐dependent [6, 24], meaning that >50% of cancer cells lacking functional p53 or other timekeeper genes do not arrest in G1 in response to prolonged mitosis. In these deficient cells, alternative pathways, such as apoptosis or mitotic slippage without G1 arrest, determine cell fate following mitotic disturbances. In p53‐deficient HeLa cells, truncated CDC20 proteoforms promote mitotic slippage, enhancing cell viability by escaping mitotic cell death mechanisms [15]. Interestingly, some cancer cells carry CDC20 mutations that alter isoform expression. An example is the endometrial adenocarcinoma cell line HEC‐6 that carries a heterozygous nonsense mutation (Q3‐stop) in *CDC20*, which eliminates full‐length CDC20 expression while increasing levels of the SAC‐resistant isoform, CDC20^M43^, translated from a downstream start codon. As a result, these cells exhibit a shortened mitotic arrest upon treatment with the Eg5 inhibitor STLC and are able to evade mitotic apoptosis when exposed to anti‐mitotic drugs [15].

## 6 Conclusion

Based on studies in yeast, it was long believed that the SAC arrests mitotically defective cells in prometaphase, providing time for error correction before the cells complete mitosis and re‐enter G1 [34, 35]. However, recent studies have revealed a more complex interplay in human cells. In p53‐proficient cells, SAC‐induced mitotic arrest is not only measurable but also inheritable. If this arrest persists beyond a critical threshold, it can trigger a G1 arrest and halt proliferation, even if chromosome segregation is ultimately successful. Ultimately, these G1‐phase cells undergo apoptosis [18].

In cells lacking functional mitotic timekeeper mechanisms, mitotic cell death can still occur in response to prolonged mitosis. However, it is important to note that these mechanisms operate on different time scales. The mitotic stopwatch is activated after a relatively short delay in mitosis (exceeding 60 min), whereas mitotic cell death mechanisms typically act much later (after 7–15 h in mitosis). Cells that escape mitotic arrest through mitotic slippage can evade mitotic cell death. This explains why cancer cells, which are prone to slippage, for instance, through expression of *CDC20^M43^*, tend to be more resistant to mitotic poisons [15].

Ultimately, cellular responses to mitosis‐targeting anti‐cancer drugs depend on both the regulation of mitotic duration and the integrity of the mitotic timekeeper machinery. The relative activity of these regulatory factors determines cell fate and resistance to drugs that target mitosis. A thorough understanding of these mechanisms across different cell types will be essential for predicting the efficacy of SAC‐activating chemotherapeutic agents.

## Author Contributions

The author takes full responsibility for this article.

## Conflicts of Interest

The authors declare no conflicts of interest.

## Data Availability

Data sharing not applicable to this article as no datasets were generated or analyzed during the current study.
